# Fluorescence in situ hybridization microscopic detection of Bacilli Calmette Guérin mycobacteria in aortic lesions: A case report

**DOI:** 10.1097/MD.0000000000011321

**Published:** 2018-07-27

**Authors:** Florent Darriet, Paola Bernioles, Ahmed Loukil, Nadia Saidani, Carole Eldin, Michel Drancourt

**Affiliations:** a Microbiology Laboratory; b Infectious Disease Department, Assistance Publique -Hôpitaux de Marseille; c Aix-Marseille University, Institut de Recherche pour le Développement, Microbes, Evolution, Phylogénie, Infections, Institut Hospitalier Universitaire Méditerranée-Infection, Marseille, France.

**Keywords:** aortic aneurysm, Bacilli Calmette Guérin, bladder cancer, fluorescence in situ hybridization, *Mycobacterium bovis*

## Abstract

**Rationale:**

To improve the diagnosis of life-threatening Bacilli Calmette Guérin (BCG) arterial aneurysm in patients treated by intravesical instillation of BCG vaccine as adjunctive therapy for non-muscular bladder carcinoma, is a life-threatening condition. Its diagnosis remains cumbersome.

**Patient concerns:**

One patient with a history of intravesical BCG installation presented with aortic aneurysm with routine microscopic examination after Ziehl-Neelsen staining remaining negative.

**Diagnoses:**

We used fluorescence in situ hybridization (FISH) to target the *Mycobacterium tuberculosis* complex *rpob* gene in a fresh aortic specimen. FISH yielded fluorescent mycobacteria in aortic lesions; mycobacteria were further confirmed as *Mycobacterium bovis* BCG mycobacteria by polymerase chain reaction (PCR) sequencing.

**Interventions:**

The patient benefited from an antituberculous treatment combining rifampicin, isoniazid, and ethambunol.

**Outcome:**

A 9-month follow-up indicated a favorable outcome.

**Lessons:**

This case report teaches that FISH targeting the *M tuberculosis* complex *rpo*B gene should be incorporated in the laboratory investigation of aortic aneurysm in patients with a history of bladder carcinoma.

## 1 Introduction

Intravesical instillation of Bacilli Calmette Guérin (BCG) vaccine has been used for 40 years as the gold standard adjunctive therapy for non-muscular bladder carcinoma.^[1]^ It is considered as a safe and well tolerated treatment achieving a 70% to 90% cure rate and decreasing recurrence and progression probabilities.^[2,3]^ The local immune response induced by BCG is complex, but presentation of mycobacterial antigen by phagocytes to T helper cells is the pivotal interaction which leads to inflammatory response efficient against tumor cells.^[4]^ BCG therapy yields transient side effects including lower urinary tract symptoms such as urgency, frequency, and dysuria in up to 50% of patients, hematuria in up to 30% of treated patients, and flu syndrome in up to 30% of patients.^[2,5]^ In addition, rare but severe side effects result from BCG systemic infection.^[6,7]^ Life-threatening BCG arterial aneurysm responsible for sudden death is known since the end of the 80s^[8,9]^ but has been reported in only 35 cases.^[10–15]^ Its laboratory diagnostic remains cumbersome and may take weeks, which are necessary for the culture of the fastidious BCG mycobacteria.

We are reporting 1 case in which first-line laboratory diagnostic was made by fluorescence in situ hybridization (FISH) microscopic detection of BCG mycobacteria in the aortic lesions, offering a new diagnostic tool to be routinely implemented. This study was approved by Méditerranée Infection Institute Ethic Comittee n 2016-024 of October 19, 2016.

## 2 Case description

**Figure 1:**
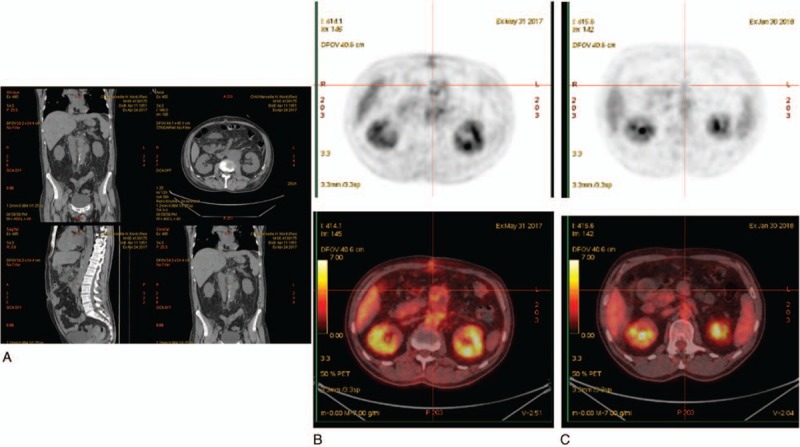
Thoraco-abdomino-pelvic computerized tomography scan showing sub-renal abdominal aorta pseudo-measuring 50 mm in the largest diameter with evidence of a collection in the left psoas muscle (A). Comparative 18FDG PET/CT before and after treatment: The 18FDG PET/CT performed before treatment showing intense hypermetabolism of the aneurysm (B) and the 18FDG PET/CT performed after treatment, showing reduction of the metabolism (C). 18FDG PET/CT = 2-deoxy-2-[fluorine-18]fluoro-D-glucose positron emission tomography combined with computed tomography.

**Figure 2:**
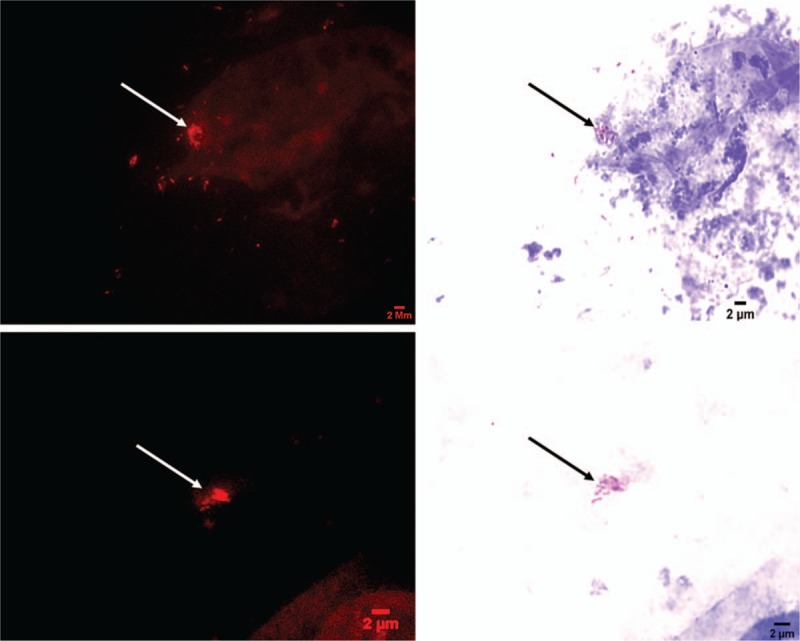
Microscopic images of an aortic aneurysm surgical specimen combining FISH (left panels) with Ziehl-Neelsen staining (right panels). The slide was observed with a Leica DMI6000 microscope under 100 times magnification using a red filter for FISH. The same microscopic field was captured using a Hamamatsu Orca AG camera (Hamamatsu Photonics, Herrsching-am-Ammersee, Germany) for FISH-positive mycobacteria (white arrows) and using a DFC425C Digital Microscope Camera (Leica Microsystèmes, Nanterre, France) for Ziehl-Neelsen-positive mycobacteria (black arrows). FISH = fluorescence in situ hybridization.

A 66-year-old man was hospitalized in April 2017 for a weight loss of 7 kg and a nocturnal low-grade fever evolving since January 2017. The patient reported clinical tuberculosis in childhood. His medical history included arterial hypertension, dyslipidemia, coronary artery disease, and tobacco smoking. A bladder cancer diagnosed in 2015 had been treated with local resection and weekly intravesical instillation of BCG (BCG-MEDAC, strain RIVM 1173-P2, MEDAC, Lyon, France) for 6 weeks. In April 2017, a thoraco-abdomino-pelvic computerized tomography scan diagnosed pulmonary embolism, a sub-renal septic aneurysm and a collection in the right psoas muscle (Fig. 1A). A 2-deoxy-2-[fluorine-18]fluoro-D-glucose positron emission tomography combined with computed tomography (18FDG PET/CT) was subsequently performed and showed an intense hypermetabolism of the aortic aneurysm, with no other embolic foci (Fig. 1B). Physical examination found dyspnea and diffuse abdominal pain. Remarkable biological parameters included hemoglobin concentration of 10.8 g/dL (normal value, 13–16 g/dL), 0.7 G/L lymphocytes (normal value, 1–4 G/L), and a C-reactive protein of 60 mg/L (normal value, 0–5 mg/L). Surgical flattening of the aneurysm was immediately performed which showed rupture on the right flank of the aorta, explaining the psoas hematoma. Postoperative probabilistic therapy included 4 g tazocillin 3 times a day and 1 bolus of 320 mg gentamicin. Routine bacteriological investigations of an aneurysm specimen collected during surgery remained negative. Pathological examination yielded chronic granulomatous inflammation of the vascular wall leading to a differential diagnosis of BCG aneurysm. While routine microscopic examination after Ziehl-Neelsen staining (Kit Quick-TB, RAL DIAGNOSTICS, Martillac, France) remained inconclusive, the surgical specimen was examined by using fluorescence in situ hybridization specifically targeting the *M tuberculosis* complex *rpo*B gene. Briefly, the fresh specimen was cut and an imprint slide was prepared by dabbing the cut surface of the biopsy against a clean glass slide after 3 washes with Dulbecco's phosphate-buffered saline (DPBS) (Thermo Fisher Scientific, Illkirch, France), then heat-fixed at 90 °C for 5 minutes and flooded with 70% ethanol for 10 minutes. The slide was covered with 10 mg/mL lysozyme (37 °C for 30 minutes), then with 5 μg/mL proteinase K (37 °C for 5 minutes) and finally with 10 μL of solution containing the rpoBMTC probe. After hybridization and appropriate washings, the slide was stained with Ziehl-Neelsen staining in a dark room and observed under 100 times magnification using a red filter and a fluorescent microscope. The overall procedure took 20 hours. Combining rpoBMTC-FISH and Ziehl-Neelsen-staining yielded specific detection of tuberculous mycobacteria as red fluorescent Ziehl-Neelsen-positive bacteria (Fig. 2). Identification at the species level was confirmed by multiplex polymerase chain reaction (PCR) amplification of regions of differences (RD) RD1, RD4, RD9, and RD12 as previously described^[16]^; and by PCR-sequencing of the *rpo*B gene which detected *M bovis* BCG with 99% sequence similarity with the reference species (GenBank AM408590.1).^[17]^ Culture for mycobacteria remained sterile. Tazocillin was discontinued and an anti-tuberculous treatment including ethambutol 500 mg 3 times a day plus an oral combination of isoniazid and rifampicin was continued for 2 months then relayed by an oral combination of rifampicin and isoniazid for 8 months. A CT scan performed at 6-month follow-up showed the absence of relapse of the aortic collection while the 18FDG PET/CT showed a reduction of the size and of the metabolism of the aortic aneurysm (Fig. 1C). A 9-month clinical follow-up indicated a favorable clinical and biological evolution.

## 3 Discussion

Urothelial non-muscle invasive bladder cancer has an average yearly incidence of 300,000 to 400,000 new cases worldwide.^[18]^ While men are 3 to 4 times more likely than women to develop non-muscle invasive bladder cancer,^[18]^ only 35 men have been reported to develop arterial aneurysms after bladder BCG instillation. The reason for this significant observation is unclear as both women and men receive huge inocula of BCG. We calculated that the case here reported received approximately 10^10^ CFUs BCG over 6 weeks, which is 10^5^ times more than the standard BCG intradermal vaccine. BCG arterial aneurysms involve the peripheral vascular system of the lower extremities,^[13–14]^ carotid arteries,^[15,19]^ and infrarenal aorta in most cases. In 1 case, BCG infection developed on a preexisting abdominal aortic aneurysm.^[20]^

BCG aneurysm is a life-threatening^[21,22]^ complication which can lead to brutal rupture of artery and sudden death. However, the mean time of diagnosis is of 23 months (range, 4–69 months) following BCG therapy.^[15]^ Once suspected, confirmation of the diagnosis exclusively relies on the accurate detection of BCG mycobacteria in the arterial lesions. In the case here reported, first line diagnostic was done by using a specific FISH protocol while routinel microscopic examination after Ziehl-Neelsen staining remained inconclusive. The result was further confirmed by detecting specific sequences using PCR sequencing. It is noteworthy that repeating Ziehl-Neelsen staining on the same slide used for FISH finally revealed acid-fast bacilli in the aneurysm lesions. It remains unclear whether this was due to the sensitizing staining effect of the FISH enzymatic treatment or increased awareness of technicians after knowing FISH positivity or both.

This observation is of interest as the review of published cases indicates that direct microscopic examination of the lesions using Ziehl-Neelsen staining failed to detect BCG arterial aneurysm in about 60% of cases.^[7]^

## 4 Conclusion

The protocol here reported should increase the ratio of microscopic diagnostic of BCG arterial aneurysm. However, microscopic examination of the lesions remains the first line diagnostic procedure as it allows visualizing the mycobacteria inside the lesions, contrary to molecular techniques

## Author contributions

**Conceptualization:** Carole Eldin, Michel Drancourt.

**Data curation:** Florent Darriet, Ahmed Loukil, Carole Eldin, Michel Drancourt.

**Formal analysis:** Florent Darriet, Ahmed Loukil, Carole Eldin, Michel Drancourt.

**Investigation:** Paola Bernioles, Ahmed Loukil, Nadia Saidani, Carole Eldin.

**Supervision:** Michel Drancourt.

**Validation:** Carole Eldin, Michel Drancourt.

**Writing – original draft:** Florent Darriet, Ahmed Loukil, Carole Eldin, Michel Drancourt.

**Writing – review and editing:** Carole Eldin, Michel Drancourt.
